# Total laparoscopic surgery for treatment of leiomyoma of the transverse colon in a patient with an abdominal mesh: A case report

**DOI:** 10.1016/j.ijscr.2019.09.043

**Published:** 2019-10-04

**Authors:** Goshi Fujimoto, Shunichi Osada

**Affiliations:** Department of Gastroenterological Surgery, Ofuna Chuo Hospital, 6-2-24, Ofuna, Kamakura, Kanagawa 247-0056, Japan

**Keywords:** TRAM flap, transverse rectus abdominis myocutaneous flap, GIST, gastrointestinal stromal tumor, NOSE, natural orifice specimen extraction, SSI, surgical site infection, Intraabdominal anastomosis, Leiomyoma, Total laparoscopic surgery, TRAM flap, Abdominal mesh, Case report

## Abstract

**Introduction:**

Gastrointestinal stromal tumors are the most common mesenchymal tumors of the gastrointestinal tract; however, gastrointestinal leiomyomas are relatively rare. Surgical resection is recommended for leiomyomas and gastrointestinal stromal tumors of the colon. We present a case in which we performed laparoscopic right hemicolectomy with intraabdominal anastomosis for treating leiomyoma of the transverse colon in a patient with an abdominal mesh.

**Presentation of case:**

A 64-year-old woman with a history of right subtotal adrenalectomy and right mastectomy was incidentally found to have an abdominal mass on a follow-up computed tomography (CT) scan, which was confirmed as a gastrointestinal stromal tumor of the mesentery following abdominal contrast-enhanced CT. We planned surgical resection for preoperative diagnosis because the tumor was >5 cm in diameter. However, she had undergone transverse rectus abdominis myocutaneous (TRAM) flap reconstruction after right mastectomy, in which the TRAM flap was replaced with an abdominal mesh; hence, total laparoscopic surgery was performed to avoid damaging the mesh owing to the risk of mesh infection. Laparotomy revealed that the tumor originated from the transverse colon; thus, transverse colectomy with intraabdominal anastomosis was performed. The total operative time and blood loss were 3 h 32 min and 5 mL, respectively. No postoperative leakage or mesh infection was observed. The resected specimen revealed a leiomyoma without malignancy.

**Discussion:**

We successfully performed colectomy that minimized the resection range and intraabdominal anastomosis.

**Conclusion:**

Total laparoscopic surgery was effective for colonic leiomyoma with an abdominal mesh to avoid mesh-related complications.

## Highlights

• Leiomyomas of the colon are rare and account for 3% of gastrointestinal leiomyomas.
• The patient had an abdominal mesh from a prior surgery.
• Laparoscopic colectomy was used for treatment of leiomyoma of the transverse colon.
• Laparoscopic colectomy prevented damage to the abdominal mesh.
• Total laparoscopic surgery is a viable option in patients with abdominal mesh.

## 1 Introduction

Mesenchymal tumors of the gastrointestinal tract are mostly gastrointestinal stromal tumors (GISTs) [1]. However, leiomyomas of the colon are rare and account for 3% of all gastrointestinal leiomyomas [2]. Since large leiomyomas have malignant potential, surgical resection is recommended for treatment, as for GISTs [3]. Benign leiomyomas can be excised locally, and laparoscopic surgery for the treatment of gastric leiomyomas, rectal GISTs, and small bowel GISTs have been reported [4,5]. Herein, we report the case of a patient with an abdominal mesh who underwent total laparoscopic colectomy for treatment of leiomyoma of the transverse colon. This work was reported in line with the SCARE criteria [6].

## 2 Presentation of case

**Fig. 1:**
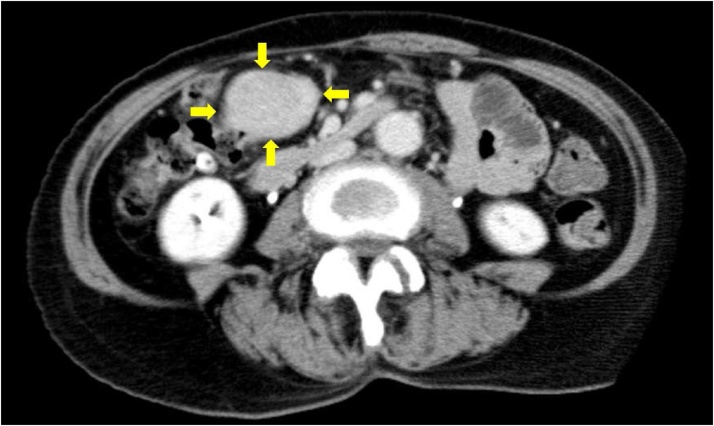
Abdominal contrast-enhanced computed tomography findings. A sharp border and homogeneous mass (arrows) are seen in the arterial phase, suggesting a gastrointestinal stromal tumor of the mesentery.

A 64-year-old woman presented with a history of right subtotal adrenalectomy for treatment of primary hyperaldosteronism and right mastectomy for treatment of breast cancer. She was incidentally detected with an abdominal mass with no abdominal symptoms during a follow-up computed tomography (CT) scan for primary hyperaldosteronism by her previous doctor. She chose to undergo surgery in our hospital where she had undergone right mastectomy and was referred to us. On physical examination, an egg-sized, elastic, soft mass with good mobility was palpable. Blood examination showed no anemia or elevation of tumor markers (Table 1). Abdominal contrast-enhanced CT revealed a sharp border and homogeneous mass, indicating GIST of the mesentery (Fig. 1). Although the patient experienced no abdominal pain, we planned surgical resection of the GIST because the tumor was over 5 cm in diameter.

**Table 1:** Results of blood examination.

Complete blood count	
WBC	3850/μL
RBC	442 × 10^4^/μL
Hb	13.0 g/dL
Ht	39.80%
Plt	22 × 10^4^/μL

Blood coagulation test	
PT (INR)	1.02
PT	96.20%
APTT	27.1 s

Serum chemistry	
TP	7.7 g/dL
Alb	4.2 g/dL
T-Bil	0.6 mg/dL
D-Bil	0.2 mg/dL
BUN	18 mg/dL
Cr	0.97 mg/dL
LDH	153 IU/L
CK	36 IU/L
AST	14 IU/L
ALT	5 IU/L
ALP	208 IU/L
γGTP	16 IU/L
Na	138 mEq/L
K	3.7 mEq/L
Cl	104 mEq/L
CRP	0.17 mg/dL
CEA	1.1 ng/dL
CA19-9	3.2 U/mL
IL-2R	253 U/mL

Alb, albumin; ALP, alkaline phosphatase; ALT, alanine aminotransferase; APTT, activated partial thrombin time; AST, aspartate aminotransferase; BUN, blood urea nitrogen; CA19-9, carcinoma antigen 19-9; CEA, carcinoembryonic antigen; CK, creatine kinase; Cl, chlorine; Cr, creatinine; CRP, C-reactive protein; D-Bil, direct bilirubin; γGTP, γ-glutamyltransferase; Hb, hemoglobin; Ht, hematocrit; IL-2R, interleukin-2 receptor; INR, international normalized ratio; K, potassium; LDH, lactate dehydrogenase; Na, sodium; Plt, platelet; PT, prothrombin time; RBC, red blood cell; T-Bil, total bilirubin; TP, total protein; WBC, white blood cell.

**Fig. 2:**
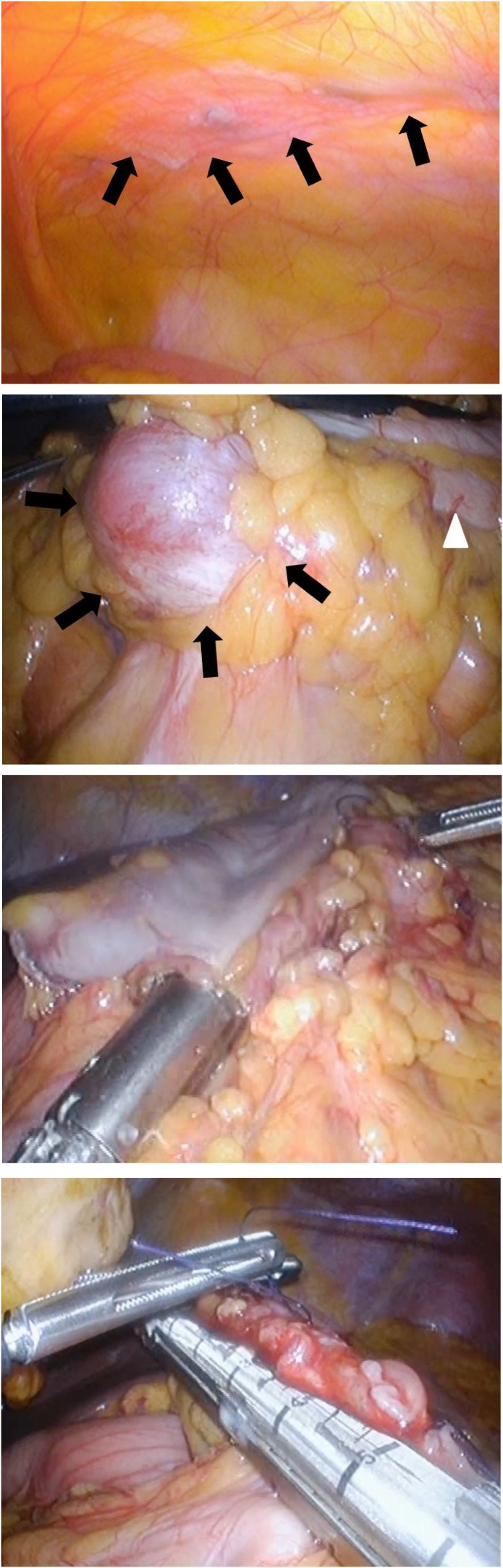
a. The abdominal mesh detected via laparoscopy. The edge of the mesh (arrows) could be detected. b. Laparoscopy findings. Laparoscopy revealed that the tumor (arrows) origin as the transverse colon (arrowheads). c. Anastomosis findings during laparotomy. The side-to-side anastomosis of the colons using a linear stapler is performed. d. Enterotomy findings during laparotomy. The enterotomy site is closed with a linear stapler by using the intraabdominal sutures as a guide.

Following previous right mastectomy, the patient had undergone transverse rectus abdominis myocutaneous (TRAM) flap reconstruction, in which her TRAM flap was replaced with an abdominal mesh. Therefore, we avoided midline abdominal incisions. We began the surgery by inserting a camera port (Kii Balloon Blunt Tip System 12 × 100 mm) into the umbilicus via an open method, as the abdominal mesh was not located around the umbilical area. After the edge of the mesh was detected with a laparoscope (Fig. 2a), we inserted two 5-mm ports (Kii Access System 5 mm) into the left and right upper quadrants, and two 12-mm ports (Kii Access System 12 mm) into the left and right lower quadrants, under a pneumoperitoneum of 10 mmHg, with a view of the laparoscopic image. Subsequently, laparotomy revealed the tumor in the transverse colon; thus, a transverse colectomy with minimal resection was planned (Fig. 2b).

After incision of the peritoneum at the base of the mesentery of the small intestine near the second portion of the duodenum, which spanned from the cranial-medial side to the caudal-lateral side, dissection between the right fusion fascia of Toldt and deep subperitoneal fascia was performed. During this lateral-to-medial-retroperitoneal dissection, the right colon was dissected freely. Adhesions of the tumor and transverse mesocolon near the superior mesenteric artery were dissected carefully, to avoid injury to marginal artery of the colon and subsequently ischemia of the transverse colon and extensive resection. Owing to the absence of lymphadenopathy, lymphadenectomy was not performed. The transverse colon was cut on the oral and anal sides of the tumor with clear margins using a linear stapler (Endo GIA™ Tristapler Purple 60 mm (Covidien)). Thereafter, the hepatic flexure of the transverse colon was moved to the left side so that it overlapped the anal side of the transverse colon, and these were fixed with stay sutures. Thereafter, a small enterotomy was made on the antimesenteric sides of these portions of the colon, and a linear stapler was inserted through the enterotomy site so that a side-to-side anastomosis could be performed (Fig. 2c). The enterotomy site was then closed roughly with three intraabdominal sutures, and using the elevation of the sutures as a guide, the site was closed completely using the linear stapler (Fig. 2d). Thus, intraabdominal anastomosis was completed. The resected specimen was retrieved through a small transverse incision made through the 12-mm port wound on the left lower region of the abdomen. The total operative time was 3 h and 32 min, and the total intraoperative blood loss was 5 mL.

**Fig. 3:**
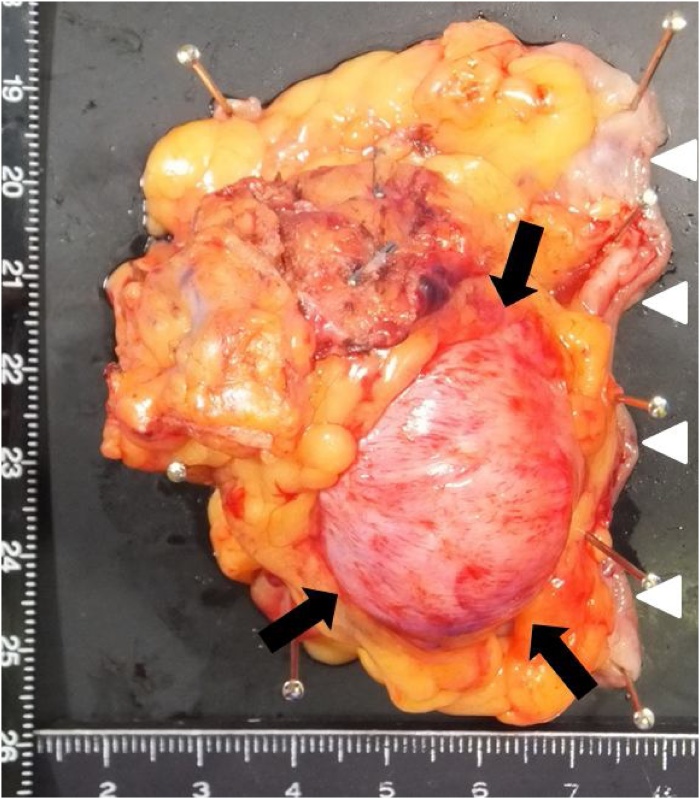

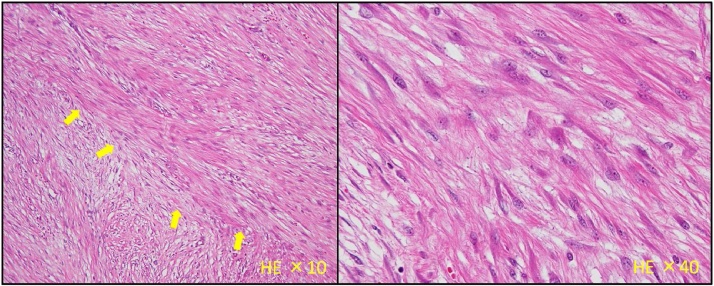
a. Macroscopic findings of the resected specimen. The resected specimen shows a smoothly marginated tumor (arrows) that is 55 × 35 × 30 mm on the serosal side (arrowheads indicate oral side of resected margin). b. Microscopic pathology findings (hematoxylin eosin stain of the resected specimen). The resected specimen shows spindle cells (arrows) in the proper muscle layer which tested positive for smooth muscle actin and negative for DOG1, CD34, desmin, CD56, CD117 (c-kit), and synaptophysin.

The resected specimen showed a tumor measuring 55 × 35 × 30 mm with smooth margins on the serosal side (Fig. 3a). Histological section of the tumor revealed a nodular lesion composed of interlacing spindle cells in the proper muscle layer. The cellularity of the tumor tissue was low, and no necrosis and nuclear pleomorphisms were observed. Upon immunostaining, the tumor was positive for smooth muscle actin and negative for DOG1, CD34, desmin, CD56, CD117(c-kit), and synaptophysin (Fig. 3b). The Ki-67 index was 1%, and less than one mitotic figure was observed per 50 high-power fields. Therefore, the tumor was diagnosed as a leiomyoma without malignancy. The patient did not experience postoperative leakage, mesh infection, metastasis, or recurrence on follow-up 6 months after surgery.

## 3 Discussion

In our report, we present the case of a patient with an abdominal mesh who underwent total laparoscopic colectomy for treatment of leiomyoma of the transverse colon while preventing damage to the mesh. Leiomyomas of the colon are relatively rare, accounting for 3% of all gastrointestinal leiomyomas [2]. Colorectal leiomyomas are distally located; leiomyomas of the transverse colon are rare and account for 2.7% of all colorectal leiomyomas [2]. Leiomyomas are mostly asymptomatic; however, abdominal pain is the most frequent symptom, followed by a palpable mass or gastrointestinal bleeding [2]. Calcification and varying degrees of necrosis or cystic changes in both benign and malignant lesions can be seen in a CT scan [7].

Leiomyomas originate from the muscularis mucosae and are less than 2 cm and are treated successfully with endoscopic resection. However, when lesions are larger than 5 cm, surgical resection is required because of the malignant potential [3]. In our case, since the preoperative diagnosis was mesenteric GIST, 1- to 2-cm resection margins and measures to avoid injury to the tumor capsule were considered. Similarly, low-grade malignant leiomyomas may be excised with a 1-cm margin of the normal tissue, and resection margins are not associated with recurrence [4,8]. Lymphadenectomy is not recommended, owing to a low risk of lymph node metastasis.

Laparoscopic surgery for GISTs and intraabdominal anastomosis for colon cancers have been reported. However, our patient’s abdominal mesh limited the laparotomy site. Therefore, we planned partial transverse colectomy with intraabdominal anastomosis, which allowed for various options regarding where the resected specimen could be taken from, and to our knowledge, this is the first report of total laparoscopic colectomy for treatment of a patient with colonic leiomyoma. For patients with right colon cancer, total laparoscopic colectomy can be described in 3 intraabdominal steps, including exposure of the vascular pedicle of the ileocolic and right colic trunk followed by complete mobilization of the right colon and resection of the colon and side-to-side mechanical anastomosis using a linear stapler [9].

The first step was not required for leiomyoma and GISTs, because lymphadenectomy was not indicated. During the second step, dissection between the right fusion fascia of Toldt and the deep subperitoneal fascia was easy to perform along the mesenteric root because this region is rich in fat and connective tissue, making it easy to maintain the ventral side of the deep subperitoneal fascia [10]. Complete mobilization of the right colon enables easy partial portion of any part of this colon. After resection of the colon, antimesenteric enterotomy was performed 10 cm distal to the stapled ends of the colon. We closed our enterotomy site with stay sutures and linear stapler because the elevation of the stay sutures allowed the anterior bowel wall to be separated from the posterior bowel wall, thereby reducing the risk of inadvertent strictures, and the linear staplers enables shorter operative time [9,11]. Furthermore, although the resected specimens are reported to be extracted through Pfannenstiel incisions, which provide less adhesion, fewer incisional hernias, and better cosmesis, we opted for a small transverse incision of the left lower region of the abdomen to avoid mesh infection [9].

When compared to laparoscopic-assisted right hemicolectomy (an intervention performed with extra-abdominal anastomosis) for treating colon cancer, total laparoscopic right hemicolectomy (an intervention performed with intraabdominal anastomosis) has resulted in similar complication rates, shorter mini laparotomy procedures, and less pain [9]. Total laparoscopic colectomy for leiomyomas and GISTs of the right colon seem to be useful because the length of the minilaparotomy can be minimal depending on the size of the tumor. Moreover, there was no statistical difference between obese and thin patients in terms of the success rate of total laparoscopic colectomy. Additionally, natural orifice specimen extraction (NOSE) has recently been reported to induce lessser pain and provide shorter hospital stay than laparoscopic-assisted right hemicolectomy, and this method may have been suitable for our patient [12]. Favorable patients for NOSE in colorectal surgery are reported to be those with a body mass index of <30, American Society of Anesthesiologists class of ≤3, and a specimen diameter of <6.5 cm; thus, our patient satisfied these conditions [13].

Leiomyomas are positive for SMA, desmin, and muscle-specific actin and negative for CD117 (c-kit), CD34, and S100 protein [3,5]. The presence of CD117 is the most specific diagnostic criterion for discriminating GISTs from leiomyomas [2]. However, distinction between benign leiomyomas and malignant leiomyosarcomas is difficult and is mainly based on the presence of necrosis, nuclear pleomorphisms, cellularity, tumor size, and number of mitotic figures [2,7].

Although there are some reports of no recurrence in colorectal leiomyoma, a recurrence rate of 2.9% for smooth muscle tumors has been reported, and all the tumors were larger than 4 cm in diameter [8]. Histologically, gastrointestinal leiomyomas that have more than two mitoses per 50 high-power fields may have malignancy potential [8]. Careful follow-up is required for cases with large tumors.

Mesh reinforcement, when repairing the abdominal donor site during a TRAM flap breast reconstruction, leads to lower abdominal hernia/bulge complication rates compared with primary closure [14]. Similarly, the 10-year cumulative recurrence rate with mesh repair for incisional hernia is reported to be lower than that of suture repair (32% versus 63%), and retrofascial preperitoneal mesh repair is superior to suture repair with regard to recurrence, even in patients with small defects [15]. Therefore, the number of patients with abdominal meshes is expected to increase. Incisional hernias occur in 2%–20% of all patients after abdominal surgery, and patients who underwent laparoscopic ventral hernia repair are reported to have 17%–25% incidence rates of requiring a subsequent abdominal operation [14,16]. The risk factors of mesh infection in ventral incisional hernia repair include prior surgical site infection (SSI) and enterotomy [17]. SSI at a subsequent laparotomy site related to intra- and preperitoneal mesh repair occurs in 16.7% of all patients, of which 9.1% require mesh removal [14]. Thus, risk factors of SSI should be avoided especially in the mesh area. SSI occurs more frequently with open colectomy than with laparoscopic colectomy [18]. In this case, a colectomy via a mini laparotomy on the mesh area could have been a risk factor for mesh infection. Furthermore, incomplete closure of the peritoneum and damage to the preperitoneal space during inguinal hernia mesh repair caused the mesh to come in contact with the intestinal tract leading to an enterocutaneous fistula [19]. Therefore, damage to the mesh during laparotomy should be avoided.

## 4 Conclusion

We could perform the minimal range of colectomy and intraabdominal anastomosis safely, even with the rare case of leiomyoma of the transverse colon. For patients with an abdominal mesh, the current surgical procedures should be improved; total laparoscopic surgery is one of the more useful options. Methods for avoiding mesh damage during mesh repair, such as the method in this report and NOSE, will be required in the future as patients with abdominal meshes increase.

## Patient perspective

The patient was concerned about whether surgery would cause mesh infection and consequently, require reoperation to remove the mesh. Therefore, she consented to this laparoscopic method for avoiding mesh damage that would reduce the risk of mesh infection. The surgery was performed without postoperative SSI, mesh infection, or anatomic leakage. The patient was informed about the regular medical follow-up.

## Availability of data and materials

The datasets supporting the conclusions of this article are included within the article.

## Funding

The research did not receive any specific grant from funding agencies in the public, commercial, or not-for-profit sectors.

## Ethical approval

This case report was approved by the Research Ethics Committee of the Ofuna Chuo Hospital (No. 2019-007).

## Consent

Written informed consent was obtained from the patient for publication of the case report and the associated images.

## Author’s contribution

Goshi Fujimoto: Responsible for performing the procedure described in the case report, concept and design of the study, acquisition of data, drafting the manuscript, revising the manuscript, and approving the final version of the manuscript.

Shunichi Osada: Responsible for assisting the surgeon for the surgical procedure described in the case report, revising the manuscript, and approving the final version of the manuscript.

## Registration of research studies

This study was registered as a case report in the UMIN Clinical Trials Registry (https://www.umin.ac.jp/ctr/) with the unique identifying number UMIN000037909.

## Guarantor

Goshi Fujimoto.

## Provenance and peer review

Not commissioned, externally peer-reviewed

## Declaration of Competing Interest

None.
